# Insular Gray Matter Volume and Objective Quality of Life in Schizophrenia

**DOI:** 10.1371/journal.pone.0142018

**Published:** 2015-11-06

**Authors:** Teruhisa Uwatoko, Miho Yoshizumi, Jun Miyata, Shiho Ubukata, Hironobu Fujiwara, Ryosaku Kawada, Manabu Kubota, Akihiko Sasamoto, Genichi Sugihara, Toshihiko Aso, Shinichi Urayama, Hidenao Fukuyama, Toshiya Murai, Hidehiko Takahashi

**Affiliations:** 1 Department of Psychiatry, Graduate School of Medicine, Kyoto University, Kyoto, Japan; 2 Kyoto University Health Service, Kyoto, Japan; 3 Human Brain Research Center, Graduate School of Medicine, Kyoto University, Kyoto, Japan; 4 Kyoto University Hospital Integrated Clinical Education Center, Kyoto, Japan; 5 Molecular Neuroimaging Program, Molecular Imaging Center, National Institute of Radiological Sciences, Chiba, Japan; Chiba University Center for Forensic Mental Health, JAPAN

## Abstract

Improving quality of life has been recognized as an important outcome for schizophrenia treatment, although the fundamental determinants are not well understood. In this study, we investigated the association between brain structural abnormalities and objective quality of life in schizophrenia patients. Thirty-three schizophrenia patients and 42 age-, sex-, and education-matched healthy participants underwent magnetic resonance imaging. The Quality of Life Scale was used to measure objective quality of life in schizophrenia patients. Voxel-based morphometry was performed to identify regional brain alterations that correlate with Quality of Life Scale score in the patient group. Schizophrenia patients showed gray matter reductions in the frontal, temporal, limbic, and subcortical regions. We then performed voxel-based multiple regression analysis in these regions to identify any correlations between regional gray matter volume and Quality of Life Scale scores. We found that among four subcategories of the scale, the Instrumental Role category score correlated with gray matter volume in the right anterior insula in schizophrenia patients. In addition, this correlation was shown to be mediated by negative symptoms. Our findings suggest that the neural basis of objective quality of life might differ topographically from that of subjective QOL in schizophrenia.

## Introduction

Improving quality of life (QOL) is considered a crucial factor in the treatment of schizophrenia [1]. Factors associated with QOL in schizophrenia, and which can serve as predictors of QOL, include depressive symptoms [2–4], adverse drug effects [5], cognitive dysfunction [6–9], occupation [10], and positive [11, 12] and negative symptoms [6, 11, 13–16], in schizophrenia.

Nevertheless, there are several inconsistencies in the results on factors influencing QOL in schizophrenia patients [17]. While some studies report weak to moderate relationships between psychiatric positive/negative symptoms and QOL [18, 19], other studies suggest that it is difficult to determine if positive/negative symptoms have significant influences on QOL [4, 11]. This inconsistency may be attributable to there being two aspects of QOL i.e., subjective and objective QOLs [6, 13, 16]. Levels of objective and subjective QOL can differ because each may be influenced by different factors: it was reported that subjective QOL might be influenced by depression [20], insight into the illness [21], and positive symptoms [11, 22], whereas objective QOL might be determined by cognitive function [23] and negative symptoms [11]. This dichotomy is simple and seems reasonable, although we need to bear in mind that the results are still controversial: for example, subjective QOL in schizophrenia is also reported to be significantly associated with negative symptoms and poor cognitive functioning [24].

Schizophrenia patients have gray matter (GM) volume reductions in specific brain regions including the insula, anterior cingulate cortex, inferior and medial frontal gyrus, hippocampus, amygdala, and thalamus [25–28]. Furthermore, some of these regional GM alterations are related to symptom severity in schizophrenia patients. For example, a large sample voxel-based morphometry (VBM) study reported correlation between volume reduction in the perisylvian region and positive symptoms [29]. Similarly, a multimodal voxelwise meta-analysis of neuroanatomical abnormalities in schizophrenia reported a significant relationship between negative symptoms and abnormalities in the medial frontal gyrus/orbitofrontal cortex/insula [25]. However, with few exceptions, little research has been performed on the neural basis impacting QOL in schizophrenia. One such study is our previous report, which found association between regional brain volume in the dorsolateral prefrontal cortex (DLPFC) and subjective QOL in schizophrenia, which is mediated by positive symptoms [12].

In consideration of this report [12], here we investigated the relationship between objective QOL, and GM alterations with an aim to illustrate the contrast of neural basis of subjective and objective QOLs in schizophrenia patients. We hypothesized that objective QOL in schizophrenia might be related to brain morphological changes, and that such a relation might be partly mediated by clinical symptoms. We also predicted that the brain regions which were related to objective and subjective QOLs may topographically differ, and that the relationships would be mediated by different psychopathology in the two QOLs. Consequently, we first examined regional brain alterations in schizophrenia that showed significant correlation with levels of objective QOL. We then examined how clinical symptoms mediate this relationship.

## Materials and Methods

### Participants

The schizophrenia group comprised 33 patients (14 female; mean age 35.7, S.D. 9.4) referred to the Department of Psychiatry, Kyoto University Hospital (Kyoto, Japan). Each patient fulfilled the criteria for schizophrenia based on the Structured Clinical Interview for Diagnostic and Statistical Manual of Mental Disorders, 4th edition (DSM-IV), patient edition (SCID-I/P) [30]. A patients’ competence to consent was confirmed by the psychiatrist in charge and double-checked by board certified senior consultant psychiatrists. Clinical symptoms were assessed using the Positive and Negative Syndrome Scale (PANSS) [31]. All patients were taking antipsychotic medication (first-generation [*N* = 3], second-generation [*N* = 24], or first and second generation [*N* = 6]). The medication dosage on the day of scanning was converted to haloperidol equivalent, according to the practice guidelines for the treatment of patients with schizophrenia [32, 33]. Participants were all physically healthy at the time of scanning. None had a history of neurological injury or disease, severe medical illness, or substance abuse that may affect brain structure and function. The comparison group comprised 42 healthy individuals (21 female; mean age 36, S.D. 7.6) that were matched to the schizophrenia group with respect to age, sex, and education level. They were also evaluated using SCID to confirm that they had no history of neurological or psychiatric disease, and no first-degree relatives had a history of psychotic episodes. Vocabulary and block design subtests from the Wechsler Adult Intelligence Scale-Revised [34] were used to estimate verbal IQ (VIQ) and performance IQ (PIQ), respectively, by transforming age-corrected scores into T-scores. The demographic and clinical data are summarized in Table 1. All of the patients participated our previous study [12]. This study was approved by the Committee on Medical Ethics of Kyoto University, and performed in accordance with The Code of Ethics of the World Medical Association. After a complete description of the study, each participant provided written informed consent.

**Table 1:** Demographic and clinical characteristics of subjects.

	HC (*N* = 42)		SC (*N* = 33)		Statistics	
	Mean	S.D.	Mean	S.D.	t	p
**Age (years)**	36	7.6	35.7	9.4	- 0.32	n.s
**Sex (male / female)**	21 / 21	-	19 / 24		0.13	n.s
**Handedness (left / right)**	3 / 39	-	3 / 30			
**Education level**	13.8	2.5	13.7	2.5	- 0.29	n.s
**VIQ**	106.6	18.4	100.5	18.3	1.43	n.s
**PIQ**	114.3	14.8	105.3	18.3	2.54	0.01*
**Duration of illness (months)**			110.5	15.8		
**Drug (mg / day, HPD equivalent)** ^a^			11.8	8.1		
**PANSS Positive**			14.0	5.7		
**PANSS Negative**			16.1	5.7		
**QLS Interpersonal relations**			23.8	9.3		
**QLS Instrumental role**			9.6	5.4		
**QLS Intrapsychic foundations**			23.9	8.5		
**QLS Common objects and activities**			8.0	1.6		
**JSQLS Psychosocial**			45.0	19.0		
**JSQLS Motivation / energy**			52.4	11.8		
**JSQLS Symptom / side effect**			25.3	18.4		

Abbreviations: SC, schizophrenia patients; HC, healthy controls; PANSS, Positive and Negative Syndrome Scale; VIQ, estimated verbal IQ obtained from the vocabulary subtask in the Wechsler Adult Intelligence Scale-Revised (WAIS-R) by transforming age-corrected scores into T-scores; PIQ, estimated performance IQ obtained from the block design subtask in the WAIS-R by transforming age-corrected scores into T-scores; QLS = The Quality of Life Scale; SQLS = Schizophrenia Quality of Life Scale.

^a^ Haloperidol equivalent was calculated according to Inagaki and Inada [32]

* two-sample *t*-test (*p* < 0.05).

### Quality of Life Scale, Japanese version (QLS)

The Quality of Life Scale (QLS), an observer-rated 21-item questionnaire, was used to assess objective QOL in schizophrenia patients [35]. Because of good reliability and validity, the scale has been widely used previously in patients with schizophrenia [1, 6, 23]. The scale is composed of four categories: (1) Intrapsychic Foundations (IF)–clinical judgment on intrapsychic elements in the dimensions of cognition, conation, and affectivity; (2) Interpersonal Relations (IP)–various aspects of interpersonal and social experience; (3) Instrumental Role (IR)–judgments on level of accomplishment and degree of underemployment, given the person’s talents and opportunities, and satisfaction; and (4) Common Objects and Activities (OA)–possession of common objects and engagement in a range of regular activities. Each item is rated on a seven-point Likert scale ranging from 0 “severe impairment of function” to 6 “normal or unimpaired functioning”. Thus, higher scores indicate higher levels of objective QOL. All data were obtained from semi-structured interviews by a trained clinician. The standardized Japanese version of QLS was used [36].

### Schizophrenia Quality of Life Scale, Japanese version (JSQLS)

The Japanese version of the Schizophrenia Quality of Life Scale (JSQLS) [37, 38] was used to assess subjective QOL of schizophrenia patients. It is a self-report 30-item questionnaire for measuring QOL specific to patients with schizophrenia. The scale is composed of three subscales: (1) psychosocial, (2) motivation/energy, and (3) symptoms/side-effects. Each of the thirty items is rated on a five-point Likert scale ranging from 0 “strongly disagree” to 4 “strongly agree”, and lower scores indicate higher levels of subjective QOL.

### MRI acquisition and pre-processing

All participants underwent magnetic resonance imaging (MRI) scans on a 3-Tesla whole-body scanner with a 40-mT/m gradient and a receiver-only 8-channel phased-array head coil (Trio, Siemens, Erlangen, Germany). The scanning parameters for three-dimensional magnetization-prepared rapid gradient-echo (3D-MPRAGE) sequences were: repetition time (TR) = 2000 ms, echo time (TE) = 4.38 ms, T1 = 990 ms, field-of-view (FOV) = 225 × 240 mm, matrix = 240 ×256, resolution = 0.9375 × 0.9375 × 1.0 mm, and 208 total axial sections without intersection gaps. MRI data were processed and analyzed using statistical parametric mapping software (SPM8; Welcome Department of Imaging Neuroscience, London, UK), with the VBM8 toolbox (http://dbm.neuro.uni-jena.de/vbm) running in Matlab 2014b (MathWorks, Natick, MA, USA). All images were tissue classified and spatially normalized to the same stereotaxic space using the diffeomorphic anatomical registration through the exponentiated Lie algebra (DARTEL) algorithm [39]. Output images of GM, white matter, and cerebrospinal fluid segment partitions were resliced into 1 × 1 × 1 mm voxels. Voxel values from segmented and normalized GM images were multiplied (modulated) by Jacobian determinants obtained from non-linear normalization steps. The resultant GM images were smoothed using Gaussian kernels of 6 mm full width at half maximum, on which all analyses were performed.

### Data analyses

#### Regional GM reductions in patients relative to controls

To identify brain regions in which schizophrenia patients showed GM volume reductions compared with controls, two-sample *t*-tests were performed in SPM8. The effects of age and gender were excluded. A statistical threshold of *p* < 0.001 (uncorrected) with an extent threshold of 20 voxels was applied [40]. The resultant image was used as an inclusion mask for the following correlational analyses.

#### Correlation of QLS scores with GM volume

To identify brain regions where GM volume reductions in schizophrenia correlate with QLS category scores, multiple regression analyses were performed in SPM8, with age and gender as nuisance covariates. In addition, further analyses were performed. First, with a statistical threshold of *p* < 0.001 (uncorrected) and an extent threshold of 20 voxels, each participant’s GM volume data for significant clusters (eigenvariates) was extracted using the Volume-Of-Interest (VOI) function in SPM8. Second, partial correlation analysis between extracted GM volume data and each QLS category score was performed using SPSS 22 (IBM Co., Armonk, NY, USA), controlling for the effects of education, IQ, illness duration, antipsychotic medication dosage at time of scanning, and positive/negative symptoms of PANSS. Statistical significance was set at *p* < 0.05. To estimate the mediation effect of clinical symptoms on the relationship between GM volume and objective QOL, a multiple mediation analysis [41] was performed for PANSS negative and positive scores using the INDIRECT macro for SPSS [42]. Standardized coefficients between GM volume and QLS category scores in the bivariate analysis (i.e., total effect, *c* coefficient) and mediation analysis (i.e., direct effect, *c*′ coefficient), their 95% confidence intervals, and the differences between *c* and *c*′ were calculated based on 1000 bootstrap samples. In these analyses, QLS category scores were included as dependent variables, extracted GM volume data as independent variables, and each positive and negative score as mediator variables. Because of the relatively small sample size and exploratory nature of this study, multiple comparison corrections were not performed across clusters and category scores of QLS and PANSS positive/negative scores. Uncorrected *p*-values of 0.05 were regarded as the statistical threshold for significance in all correlational analyses.

### Correlational analyses between QLS and SQLS

We conducted correlational analyses between each of the QLS subcategory scores and each of SQLS subscores.

## Results

### Demographic and clinical data

Table 1 shows demographic and clinical information. There were no significant differences between patients and controls in any demographics (age, gender, handedness, and education level), except for estimated PIQ (*p* = 0.01).

### Regional GM reductions in patients relative to controls

**Fig 1:**
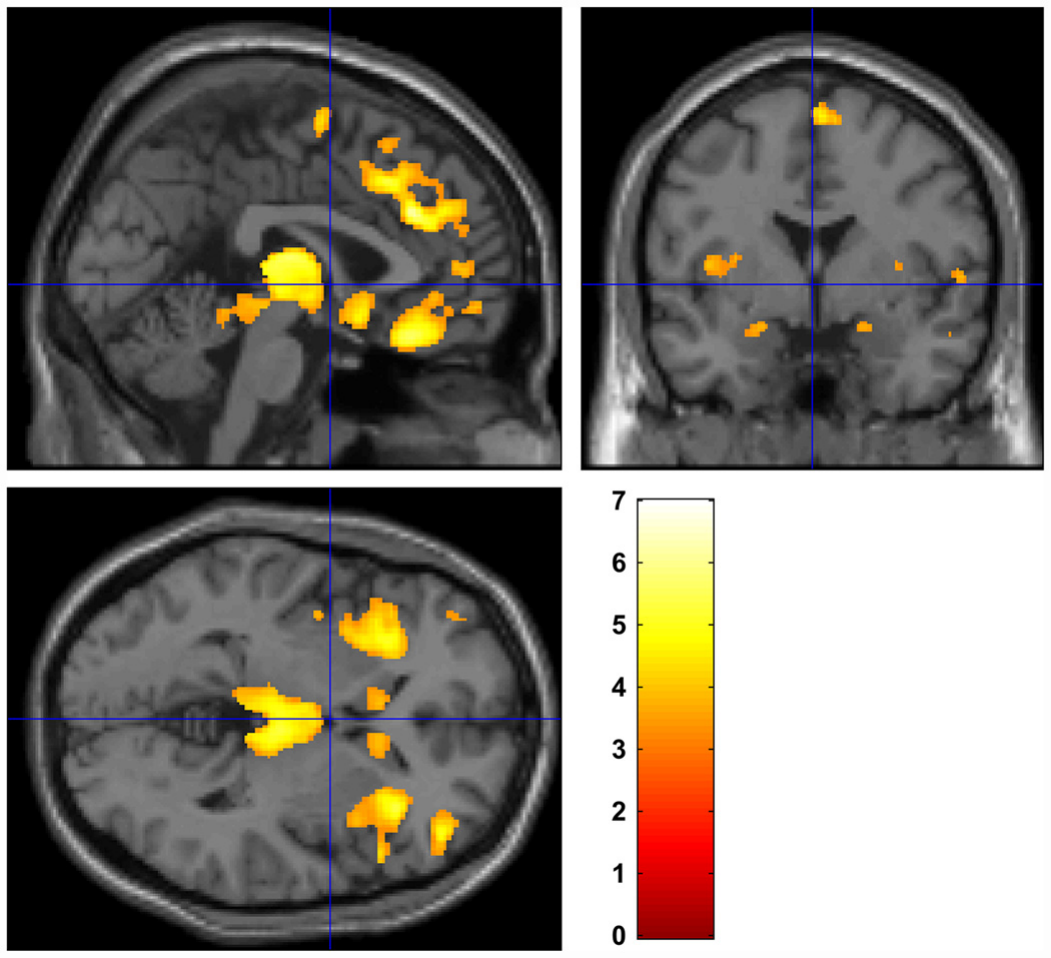
Group difference in gray matter volume. Yellow clusters reflect gray matter reductions in the inferior frontal gyrus, middle frontal gyrus, superior frontal gyrus and insula in schizophrenia patients compared with healthy controls. The color bar represents the T-score: white indicates higher statistical significance than yellow or red. Uncorrected *p* < 0.001; extent threshold 20 voxels.

In agreement with previous studies [25–28], schizophrenia patients showed GM reductions compared with controls in areas containing regions such as the bilateral inferior frontal gyrus, middle frontal gyrus, superior frontal gyrus, temporal gyrus, thalamus, and insula (Fig 1).

### Correlation analyses for GM volume and QLS category scores

**Fig 2:**
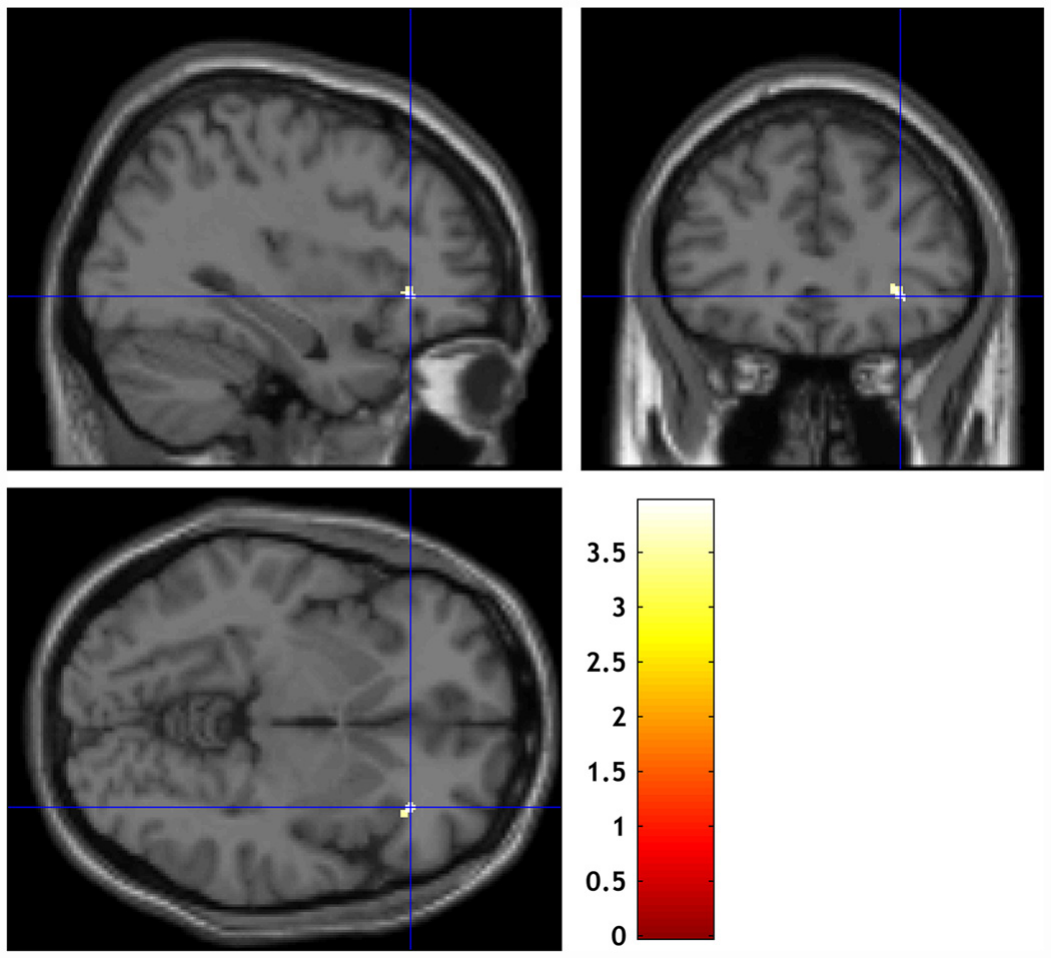
Multiple regression analysis using voxel-based morphometry to identify the brain region that significantly correlates with QLS Instrumental Role score. The white cluster on the right AI (x = 34, y = 32, z = −5; cluster size = 33) significantly correlates with QLS Instrumental Role score. The color bar represents the T-score: white indicates higher statistical significance than yellow or red. Uncorrected *p* < 0.001; extent threshold 20 voxels.

**Fig 3:**
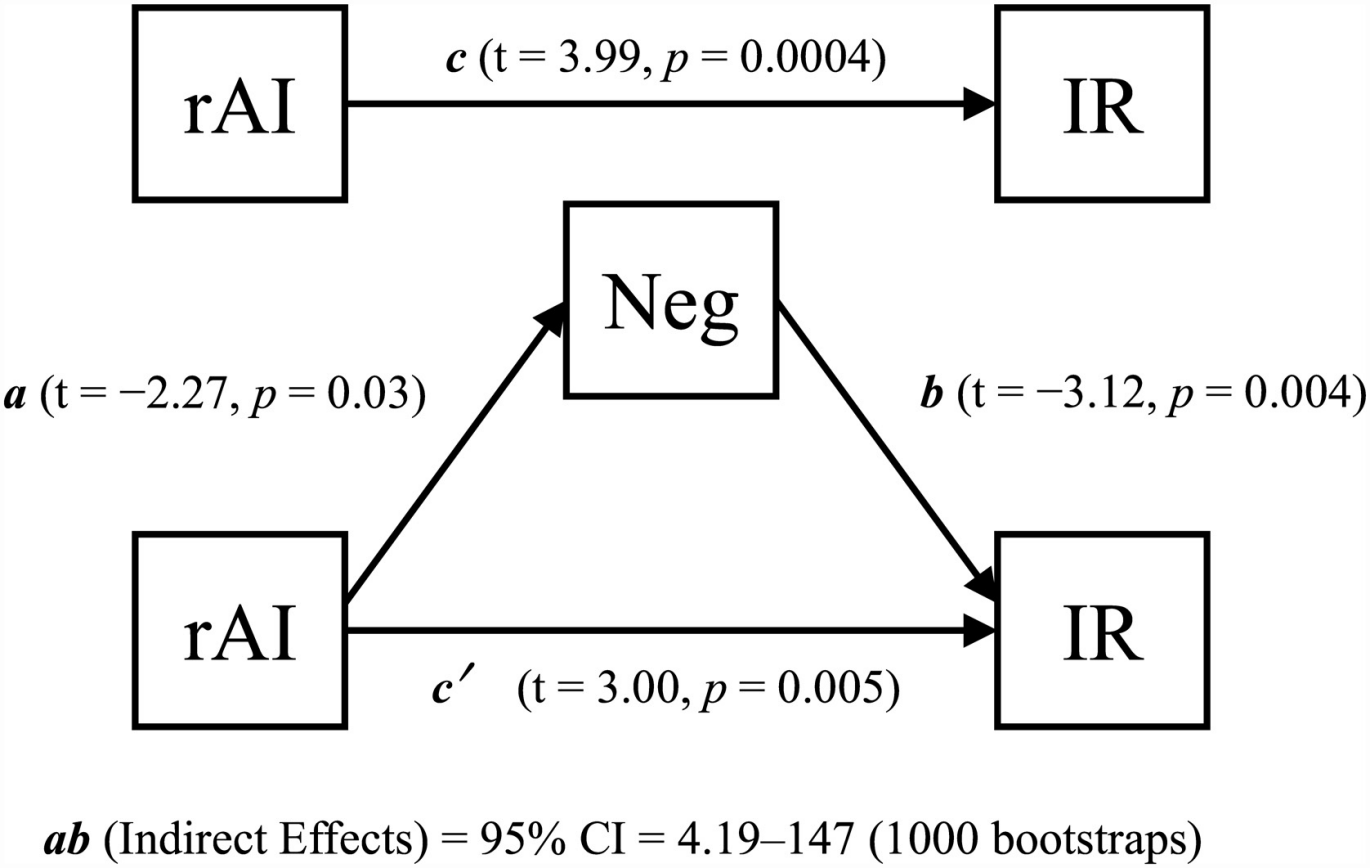
Partial mediation effect of negative symptoms on right anterior insula volume and QLS Instrumental Role score. Mediation analysis using negative symptoms as mediator. *a*: coefficient for the association between right AI volume and negative symptoms; *b*: coefficient for the association between negative symptoms and QLS Instrumental Role score; *c*: total effect from bivariate analysis; *c*′: direct effect from mediation analysis; rAI: right anterior insula gray matter volume; IR: Instrumental Role QLS category score; Neg: PANSS negative symptom score.

**Fig 4:**
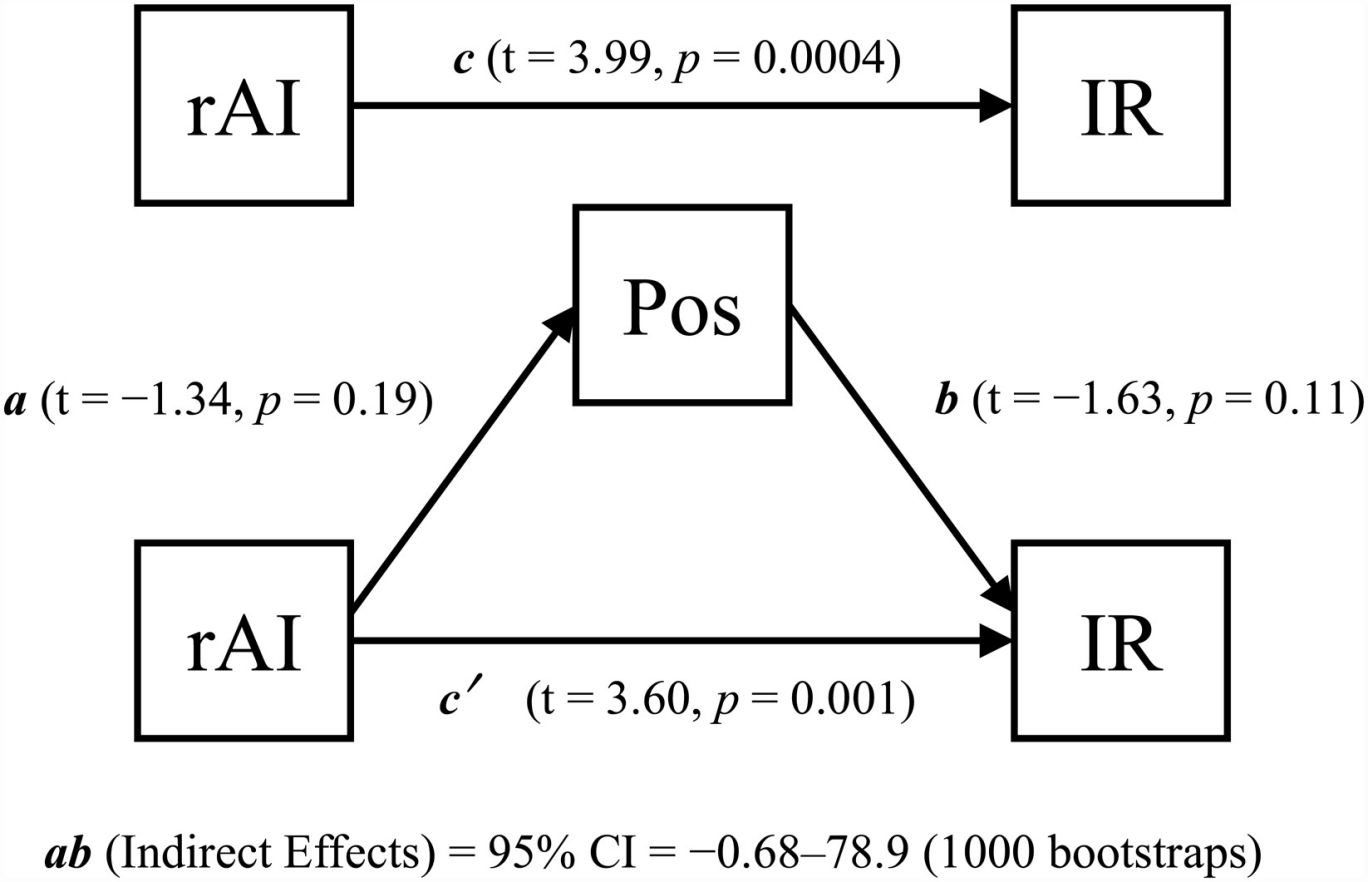
No mediation effect of positive symptoms on right AI volume and QLS Instrumental Role score. Mediation analysis using positive symptoms as the mediator. *a*: unstandardized regression coefficient for the association between right AI volume and positive symptoms; *b*: unstandardized regression coefficient for the association between negative symptoms and QLS Instrumental Role score; *c*: total effect from bivariate analysis; *c*′: direct effect from mediation analysis; rAI: right anterior insula gray matter volume; IR: Instrumental Role QLS category score; Pos: PANSS positive symptom score.

Multiple regression analyses found that IR score positively correlated with right anterior insula (AI) GM volume (peak coordinates: x, y, z = 34, 32, −5 cluster size = 33 voxels) in the patient group (Fig 2). None of the other QLS category scores significantly correlated with GM volume. Correlation between IR category score and extracted right AI volume remained significant after controlling for all demographic and clinical variables (Table 2). A specific, significant mediation effect was observed for PANSS negative score (bias corrected 95% confidence interval = 4.19–147 by 1000 bootstrap resamples) between right AI GM volume and IR score (Fig 3). The mediation effect was not significant for positive symptoms (bias corrected 95% confidence intervals = −0.68–78.9 by 1000 bootstrap resamples) (Fig 4).

**Table 2:** Correlation between right AI volume and QLS Instrumental Role (IR) category score.

AI Volume / IR Score	+ Control variables	Partial Correlation
**#0**	None	0.58***
**#1**	#0 + Education, VIQ, PIQ	0.64***
**#2**	#1 + Drug, Duration of illness	0.59**
**#3**	#2 + PANSS Positive	0.59**
**#4**	#3 + PANSS Negative	0.48*

Significant correlation at the

*** 0.001 level;

** 0.005 level; and

* 0.05 level (two-tailed).

#### Correlational analyses between QLS and JSQLS

Correlational analyses found that several JSQLS subscores were significantly correlated with subscales of objective QOL (Table 3). However, we found no correlation between each of JSQLS subscale score and IR score.

**Table 3:** Correlations between QLS and JSQLS.

	JSQLS Psychosocial	JSQLS Motivation / energy	JSQLS Symptoms / side-effects
**QLS Intrapsychic foundations**	- 0.480**	- 0.357*	- 0.389*
**QLS Interpersonal relations**	- 0.475**	- 0.306	- 0.256
**QLS Instrumental role**	- 0.236	- 0.123	- 0.089
**QLS Common objects and activities**	- 0.403*	- 0.330	- 0.390*

Significant correlation at the

** 0.01 level and

* 0.05(two-tailed).

## Discussion

To our knowledge, this is the first study to investigate the relationship between objective QOL, symptoms, and regional brain volume in schizophrenia patients. We found a significant correlation between right AI volume and objective QOL in schizophrenia patients. Furthermore, this correlation was mediated by negative symptoms, but not positive symptoms. These findings suggest that reduced GM volume in the right AI is linked to poor objective QOL in schizophrenia, and can be partially explained by negative symptoms. Our exploratory study implies that a combination of imaging and psychosocial assessments would be useful to determine the factors affecting QOL in schizophrenia.

In the patient group, right AI volume correlated with IR score, a subscale that measures sufficiency for an occupational role in social life. This correlation was not diminished even after controlling for clinical score. The insula is a brain region related to emotional processing and sensory stimuli. Within the insula, the anterior part is involved in emotional processing such as facial affect processing, and empathy [43–45], whereas the posterior part is involved in the encoding of multimodal sensory processing such as interoceptive awareness and somatosensory processing [46]. Brain alterations in the AI may be linked to dysfunction in emotional processing such as facial affect recognition and empathic ability, which underlie negative symptoms [47, 48]. This in turn, is crucial for playing a sufficient and satisfied occupational role as a member of the community [49], i.e., one aspect of objective QOL.

Mediation analysis showed that negative but not positive symptoms mediated the correlation between right AI volume and IR score. A recent study reported a relationship between negative symptoms and objective QOL in schizophrenia patients [50]. The same study also suggested that negative symptoms have a greater impact on real-world functioning than positive symptoms. Our findings are in agreement with this. Although structural and functional alterations in the insula are linked to positive symptoms in schizophrenia [51–53], recent studies on functional connectivity in schizophrenia suggest that AI dysfunction is related to negative symptoms [54, 55]. These results support our finding of a significant correlation between AI volume and negative symptoms.

The mediation model using positive symptoms as the mediator between AI and IR did not fit the data. This indicates that positive symptoms have no mediator effect on the correlation. This finding corresponds with previous studies, which report that the effect of negative symptoms on objective QOL and social functioning are more robust than those of positive symptoms [21, 50].

Correlation analyses demonstrated significant correlation between subscales of subjective and objective QOL. However, although IR score significantly correlated with AI GM volume in our study, it had no correlation with any subjective QOL subscale. Lack of correlation between IR score and objective or subjective QOL subscores was also reported previously [13]. These findings imply that at least in some part the objective and subjective QOL consists of different factors, and that combination of both of QOLs is necessary to adequately evaluate the overall picture of a patient's QOL.

In our previous study, we demonstrated correlation between *subjective* QOL and DLPFC GM volume reduction, which was mediated by positive symptoms of schizophrenia [12]. Taken this together with the current results, it is suggested that subjective and objective QOL in schizophrenia have different underlying brain factors, i.e., DLPFC and AI, respectively. In addition, links between both aspects of QOL and brain alterations are mediated by distinct clinical symptoms, i.e., positive and negative symptoms. This may explain the discrepancy between subjective and objective QOL in schizophrenia.

There are several limitations to our study. First, because all the patients were on medication, we cannot exclude the influence of adverse effects (e.g., tardive dyskinesia and extrapyramidal symptoms) on objective QOL [6, 14]. Second, we did not detect any significant correlation between regional brain volume and any other objective QOL subscale. This may be due to the small sample size, which may have resulted in loss of statistical power to detect other brain regions that correlated with each scale. In fact, with a more liberal threshold, we found correlations between each of other subcategories and regional brain volume in orbitofrontal cortex, insula, and anterior cingulate cortex. Finally, objective QOL can be affected by factors such as socioeconomic status [56] and cognitive function [23, 57], which were not assessed in this study. Thus, deliberate consideration is needed for generalization of our results.

In conclusion, we have demonstrated that the instrumental role domain of objective QOL correlates with GM alterations in the right AI in schizophrenia, and that negative symptoms play a crucial role in the relationship between objective QOL and regional brain alterations. The findings suggest that the neural basis of objective QOL might topographically differ from that of subjective QOL. Combining imaging techniques with psychosocial methods will help elucidate the factors associated with QOL, an important outcome of schizophrenia treatment.
